# Antioxidant Properties of Buffalo-Milk Dairy Products: A β-Lg Peptide Released after Gastrointestinal Digestion of Buffalo Ricotta Cheese Reduces Oxidative Stress in Intestinal Epithelial Cells

**DOI:** 10.3390/ijms19071955

**Published:** 2018-07-04

**Authors:** Manuela Giovanna Basilicata, Giacomo Pepe, Simona Adesso, Carmine Ostacolo, Marina Sala, Eduardo Sommella, Maria Carmina Scala, Antonella Messore, Giuseppina Autore, Stefania Marzocco, Pietro Campiglia

**Affiliations:** 1 Department of Pharmacy, School of Pharmacy, University of Salerno, Via Giovanni Paolo II 132, I-84084 Fisciano, Italy; mbasilicata@unisa.it (M.G.B.); gipepe@unisa.it (G.P.); sadesso@unisa.it (S.A.); msala@unisa.it (M.S.); esommella@unisa.it (E.S.); mscala@unisa.it (M.C.S.); autore@unisa.it (G.A.); 2 PhD Program in Drug Discovery and Development, University of Salerno, Via Giovanni Paolo II 132, I-84084 Fisciano, Italy; 3 Department of Pharmacy, University of Naples Federico II, Via D. Montesano 49, I-80131 Napoli, Italy; ostacolo@unina.it; 4 Dipartimento di Chimica e Tecnologie del Farmaco, Istituto Pasteur-Fondazione Cenci Bolognetti, “Sapienza” Università di Roma, P.le Aldo Moro 5, I-00185 Roma, Italy; antonella.messore@uniroma1.it; 5 European Biomedical Research Institute of Salerno, Via De Renzi 50, I-84125 Salerno, Italy

**Keywords:** buffalo-milk dairy products, in vitro gastrointestinal digestion, solid-phase synthesis (SPPS), intestinal epithelial cells (IEC-6), reactive oxygen species (ROS), NAD(P)H: quinone oxidoreductase 1 (NQO1), heme oxygenase 1 (HO-1), superoxide dismutase (SOD), nuclear factor erythroid 2-related factor 2 (Nrf2)

## Abstract

Redox signaling regulates different gastrointestinal (G.I.) epithelium functions. At the intestinal level, the loss of redox homeostasis in intestinal epithelial cells (IECs) is responsible for the pathogenesis and development of a wide diversity of G.I. disorders. Thus, the manipulation of oxidative stress in IECs could represent an important pharmacological target for different diseases. In this study, peptides released from in vitro gastro intestinal digestion of different buffalo-milk commercial dairy products were identified and evaluated for their bioactive properties. In particular, six G.I. digests of dairy products were tested in a model of oxidative stress for IECs. Among them, buffalo ricotta cheese was the most active and the presence of an abundant β-lactoglobulin peptide (YVEELKPTPEGDL, f:60-72) was also revealed. The antioxidant potential of the identified peptide was also evaluated in a model of hydrogen peroxide (H_2_O_2_)-induced oxidative stress in the IEC-6 cell line. The peptide was able to reduce ROS release, while, on the other hand, it increased nuclear factor (erythroid-derived 2)-like 2 (Nrf2) activation and the expression of antioxidant cytoprotective factors, such as heme oxygenase 1 (HO-1), NAD(P)H:quinone oxidoreductase 1 (NQO1), and superoxide dismutase (SOD). These results indicate that buffalo ricotta cheese-isolated peptide could have potential in the treatment of some gastrointestinal disorders.

## 1. Introduction

Reactive oxygen species (ROS) are byproducts of normal cellular metabolism. Low and moderate amounts of ROS have beneficial effects on several physiological processes, including killing of invading pathogens, wound healing, and tissue repair processes. However, excessive levels of ROS lead to cell damage and apoptosis and play an important role in cancer, neurodegenerative disorders, and coronary heart and inflammatory bowel diseases [1]. In particular, the gastrointestinal tract is constantly exposed to reactive oxygen species, since the presence of oxygen, acidic pH, and H_2_O_2_ in the gastric environment promote the Fenton reaction, generating superoxide anions and hydroxyl radicals. An excessive ROS production in the gastrointestinal tract damages cytoskeletal proteins and disrupts the intestinal barrier to increase gut permeability, which contributes to inflammation in a variety of gastrointestinal diseases, such as gastroduodenal and chronic intestinal inflammation (as IBD), ulceration, and gastric cancer [2,3,4].

In order to prevent and counteract these effects, the employment of antioxidant molecules is crucial. However, the use of pharmaceutical drugs is sometimes associated with side effects. For this reason, the search for natural alternatives originating from foods/dietary compounds has gained increasing attention [5]. Different studies have suggested that bioactive peptides, released from dietary proteins during digestion, could exert metabolic and physiologic actions by acting on specific targets at the digestive level or after absorption [6,7,8]. Many food proteins possess antioxidant peptide sequences which are released during the gastrointestinal digestion process. The digestion by gastrointestinal enzymes is a natural process for the release of antioxidant peptides, differing from oral administration of commercially available bioactive peptides which are subjected to degradation and, hence, inactivation, after oral intake. Furthermore, an important aspect is the low bioavailability of food-derived antioxidant peptides, which determine their accumulation in the gastrointestinal tract, suggesting that the major physiological effects could be locally explicated [9]. The presence of antioxidant peptide segments in proteins may help to explain why dietary protein intake can promote animal and human health beyond the normal nutritional benefits exerted. During gastrointestinal digestion of parent proteins there is a slow and continuous release of antioxidant peptides and amino acids, which protect the gastrointestinal tract itself and prevent the onset of oxidative stress [9].

Several studies showed many bioactive peptides in different dairy species, such as bovine, ovine, and caprine milk [10,11,12], but few studies have been conducted on buffalo-milk dairy products. Buffalo milk contributes to 13% of the total milk production in the world and is produced abundantly in regions of Southern Italy, particularly in Campania, where the buffalo find a favorable environment [13]. Buffalo milk is highly suitable for the manufacturing of a wide range of value-added dairy products [14].

In this regard, the aim of our work was to investigate the release after an in vitro gastrointestinal digestion of potential antioxidant peptides from buffalo-milk dairy products. In detail, the crude digests were tested in intestinal epithelial cell line (IEC-6), treated with H_2_O_2_, in order to evaluate the inhibition of ROS release. Buffalo ricotta cheese resulted the most active. Thus, it was fractionated by ultra-filtration using a membrane with an M.W. cutoff of 1000 Da. Peptides in the most active fraction were subjected to UHPLC-MS/MS analysis, revealing the presence of an abundant β-lactoglobulin peptide. Finally, this peptide was synthesized and tested in vitro for the evaluation of potential intestinal protection in IEC-6 treated with H_2_O_2_.

## 2. Results

### 2.1. Antioxidant Effect of G.I. Digests on the ROS Release in H_2_O_2_-Treated IEC-6 Cells

**Figure 1:**
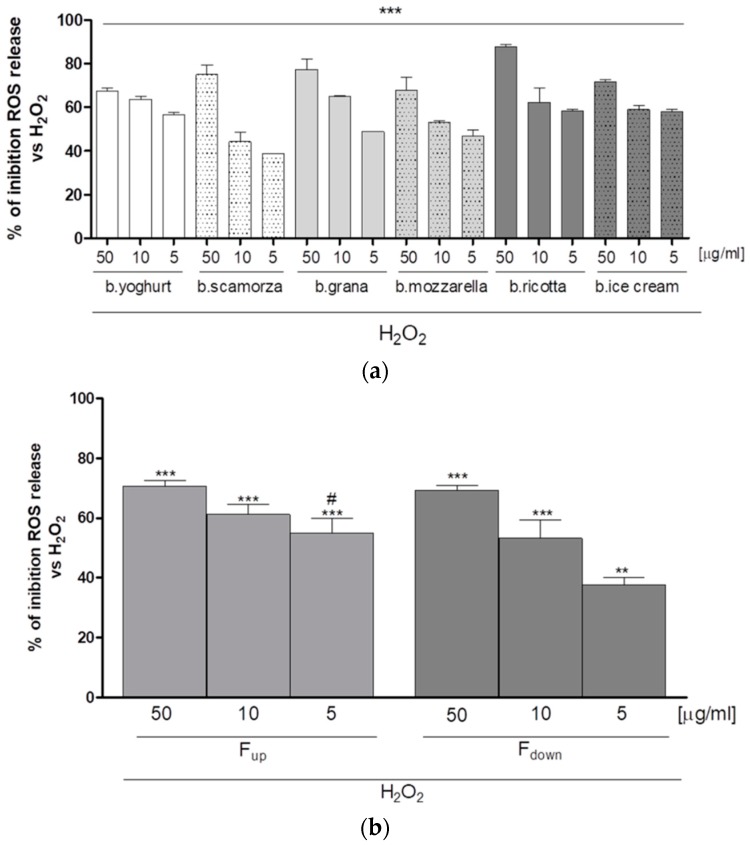
Effect on ROS formation, in IEC-6 cells, evaluated with the probe 2′,7′ dichlorofluorescein-diacetate of buffalo-milk dairy products (**a**) and buffalo ricotta peptide fractions, F_up_ and F_down_ (**b**). Values, mean ± s.e.m., are expressed as the % of inhibition of ROS release vs. H_2_O_2_. *** and ** denote *p* < 0.001 and *p* < 0.01 vs. H_2_O_2_ and ^#^ denotes *p* < 0.05 vs. F_down_.

A simulated gastrointestinal digestion of commercial buffalo-milk dairy products was carried out. Experimental conditions revealed the milk parent proteins denaturation and the generation of different peptides by action of the stomach and small intestine proteolytic enzymes. After preliminary filtration using centrifugal filter devices with 3000 NMWL (nominal molecular weight limit) and purification by SPE, the effect of G.I. digests of buffalo-milk dairy products on oxidative stress induced by H_2_O_2_ in the IEC-6 cells was evaluated. No cytotoxic effects were observed when IEC-6 cells were treated with the six crude digests at the concentrations of 5, 10, and 50 μg/mL (data not shown). Contrariwise, all tested G.I. digests induced a significant decrease of ROS release in IEC-6 treated with H_2_O_2_ (1 mM) at all tested concentrations (50–5 µg/mL, *p* < 0.001 vs. H_2_O_2_; Figure 1a). In particular, among all tested dairy commercial products, it can be appreciated how the buffalo ricotta sample showed the highest inhibitory activity against ROS release (Figure 1a). The intestinal digesta of buffalo ricotta cheese was further purified by ultra-filtration using a membrane with 1000 NMWL obtaining two fractions. Fractions both (50–5 µg/mL) have been tested for their antiproliferative activity on IEC-6 cells, after 24 h of treatment assessed by MTT assay. Our data did not show any significant cytotoxic effect for all tested concentrations extracts (data not shown). The buffalo ricotta fractions significantly inhibited ROS release in a concentration dependent manner (*p* < 0.001 vs. H_2_O_2_*;* Figure 1b) but, in particular, F_up_ showed higher activity at the lowest concentration tested (5 μg/mL) (*p* < 0.05 vs. F_down_; Figure 1b).

### 2.2. Isolation and Identification of Buffalo Ricotta Peptide (BRP)

**Figure 2:**
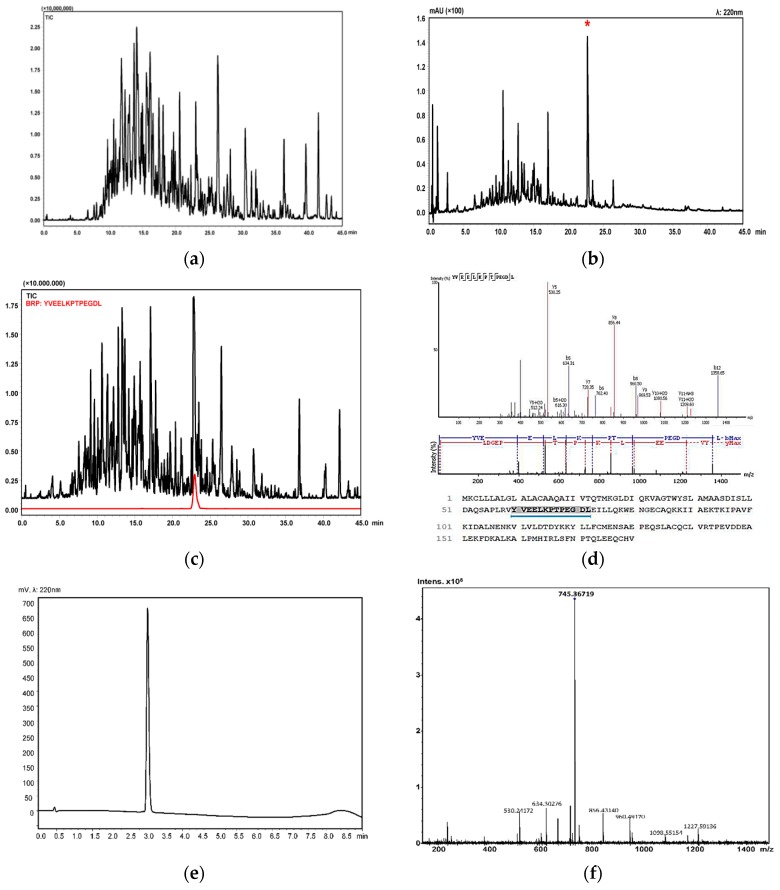
(**a**) Total ion chromatogram (TIC) of peptides released during in vitro gastrointestinal digestion of buffalo ricotta cheese; (**b**) chromatographic profile and (**c**) TIC of peptide fraction with molecular weights between 1 and 3 kDa (F_up_); (**d**) fragmentation pattern of BRP; (**e**) chromatogram acquired by RP-UHPLC-UV; and (**f**) mass spectrum of the synthetic BRP obtained by direct infusion Fourier transform ion cyclotron resonance MS.

The intestinal digesta of buffalo ricotta cheese was monitored by LC-MS/MS. As shown in Figure 2a, a high complex analytical profile was obtained. We have previously reported the complete list of identified peptides, including retention times, peptide sequences, precursor proteins, positions, and masses [15]. In order to identify the antioxidant peptides, our attention was, thus, focused on the characterization of the most abundant peptides in the fraction F_up_ by RP-UHPLC-PDA-ESI-IT-TOF. An intense peak possessing the highest area percent in the UV chromatogram, was selected (Figure 2b) and its primary structure was tentatively identified (YVEELKPTPEGDL, β-Lg f60-72, Figure 2c,d). To confirm the hypothesized chemical structure and study its specific biological activity, the peptide was synthesized by conventional solid-phase peptide synthesis methods. Analytical purity of synthetic peptide was determined by UHPLC-UV (Figure 2e) while its identity was confirmed by high-resolution MS data (Figure 2f).

### 2.3. Investigation of Antioxidant Effect of BRP in H_2_O_2_-Treated IEC-6 Cells

**Figure 3:**
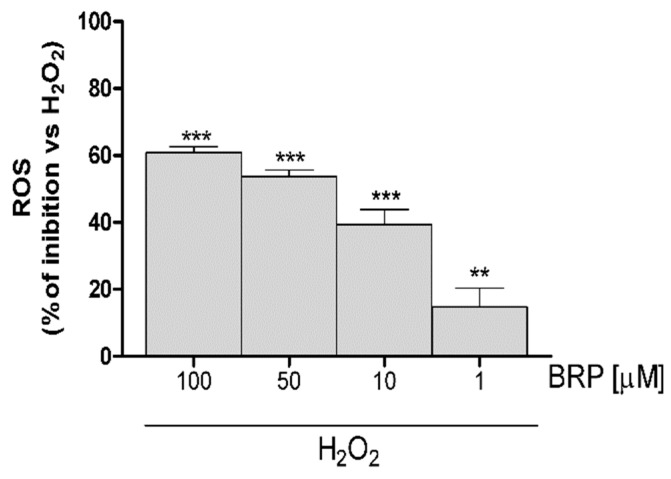
Effect of BRP on ROS formation, in IEC-6 cells. Values, mean ± s.e.m., are expressed as the % of inhibition of ROS release vs. H_2_O_2_. *** and ** denote *p* < 0.001 and *p* < 0.01 vs. H_2_O_2_.

In order to investigate the effect BRP on oxidative stress induced by H_2_O_2_ in the IEC-6 cells, the intracellular ROS production was evaluated. No significant cytotoxic effects were observed when IEC-6 cells were treated with BPR at all tested concentrations (data not shown). Our results showed that BRP produced, at all tested concentrations (100–1 µM), a significant inhibition in ROS release induced by H_2_O_2_ (1 mM; *p* < 0.01 vs. H_2_O_2_, Figure 3).

**Figure 4:**
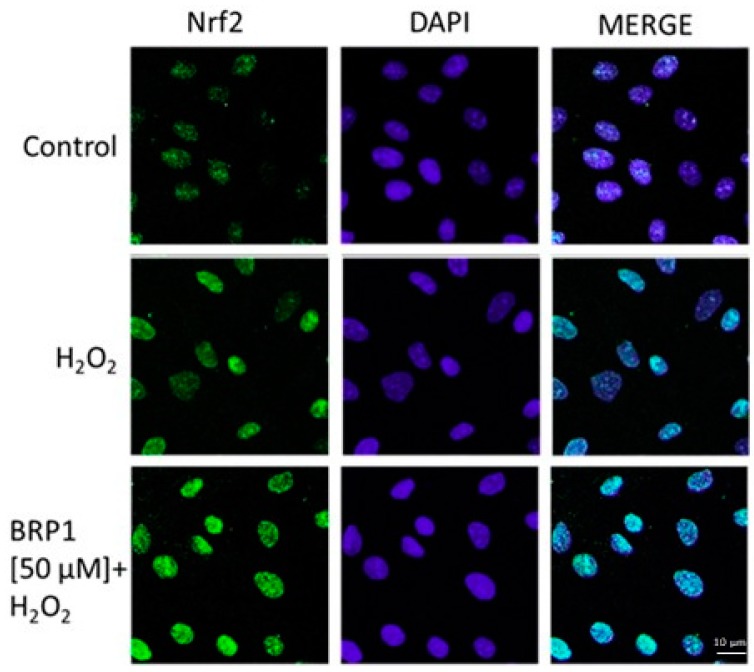
Effect of BRP (50 μM) on Nrf2 nuclear translocation, evaluated using immunofluorescence assay confocal microscopy. Scale bar: 10 μm. Blue and green fluorescence indicate the localization of nucleus (DAPI) and Nrf2, respectively.

Free radical detoxification, which is essential to reduce ROS-induced cell injury, is mediated by multiple well-coordinated antioxidant enzymes that are responsible for maintaining redox homeostasis balance. ROS exposure stimulates cells to increase the expression of antioxidant and cytoprotective enzymes. Nuclear factor erythroid 2-related factor 2 (Nrf2) is the master regulator of the cellular response to excess ROS [16]. Nrf2 is an intracellular transcription factor that regulates the expression of a number of genes to encode anti-oxidative enzymes, detoxifying factors, anti-apoptotic proteins, and drug transporters. For these reasons, in order to evaluate the antioxidant effect of the β-lactoglobulin-derived peptide, the influence on this specific antioxidant pathway was also studied. In detail, to track the influence of BRP on the antioxidant cellular response, we evaluated Nrf2 activation, labelling Nrf2 with a green fluorescent probe. As can be observed in Figure 4, a significative increase of nuclear Nrf2 was detected in IEC-6 cells treated with BRP (50 µM), with respect to cells treated with H_2_O_2_ alone (1 mM).

**Figure 5:**
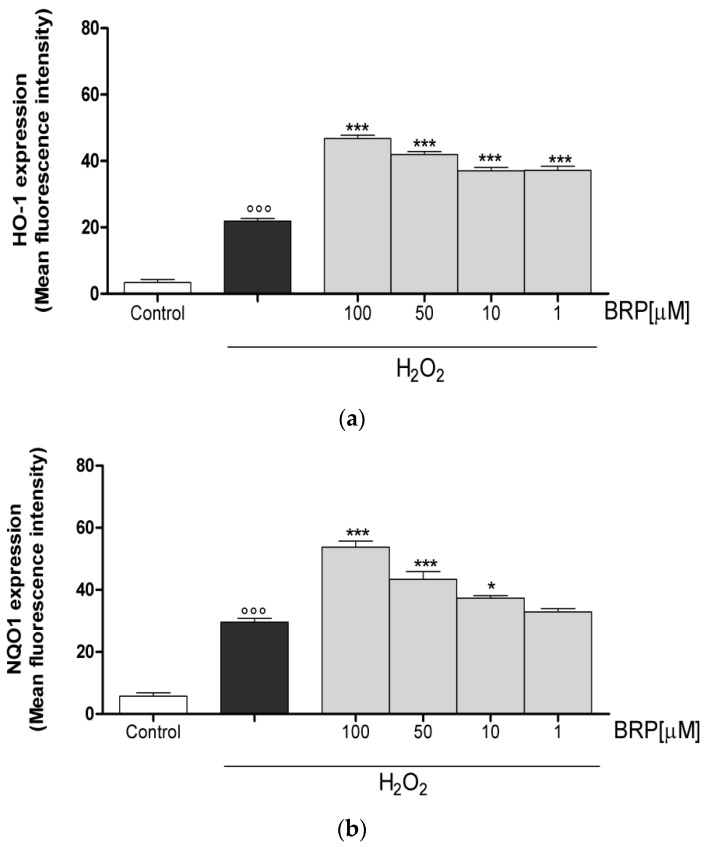

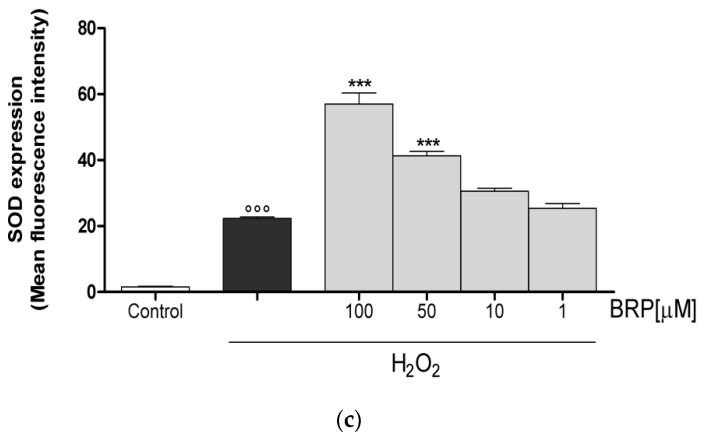
(**a**) Effect on HO-1, (**b**) NQO1, and (**c**) SOD expression of BRP in the IEC-6 cells, evaluated by cytofluorimetric technique. Values, mean ± s.e.m., are expressed as the % of inhibition of HO-1, NQO1, and SOD expression vs. H_2_O_2_ alone-treated cells. *** and * denote *p* < 0.001 and *p* < 0.05 vs. H_2_O_2_. °°° denotes *p* < 0.001 vs. control.

The activation of Nrf2 pathway leads to the expression of cytoprotective enzymes, such as NAD(P)H: quinone oxidoreductase 1 (NQO1), heme oxygenase 1 (HO-1), and superoxide dismutase (SOD) [17]. In detail, NQO1 is multifunctional antioxidant flavoprotein that catalyzes the reduction of quinones, quinoneimines, nitroaromatics, and azo dyes [18]. HO-1 is the rate limiting enzyme in the conversion of heme into biliverdin/bilirubin, iron, and carbon monoxide [19]. SOD enzymes catalyze the dismutation of superoxide radical into hydrogen peroxide and molecular oxygen and, consequently, present an important defense mechanism against superoxide radical toxicity [20]. In this context, the influence of BRP on the expression of HO-1, NQO1 and SOD enzymes was evaluated. In particular, the expression of cytoprotective enzymes significantly increases in presence of H_2_O_2_ (1 mM; *p* < 0.001 vs. control; Figure 5). When BRP (100–1 µM) was added to IEC-6 cells, a further increase in HO-1, NQO1, and SOD was observed (*p* < 0.05 vs. H_2_O_2_; Figure 5).

## 3. Discussion

The gastrointestinal tract is continuously exposed to xenobiotics, which are absorbed by the intestinal lumen and subsequently distributed into the systemic circulation. The digestive tract is equipped with defense mechanisms to detoxify reactive intermediates and minimize oxidative stress. Metabolic enzymes often convert these xenobiotics into less toxic and more water-soluble forms but, in some cases, their metabolism generates more toxic species making the gastrointestinal tract particularly susceptible to oxidative-type diseases [21].

Since the gastrointestinal tract is in contact with digested food proteins and the milk proteins can be considered as a carrier for the delivery of antioxidant peptides, in this paper the potential intestinal protection against induced oxidative stress exerted by buffalo’s milk dairy products after simulated gastrointestinal digestion was analyzed. Among all tested buffalo-milk dairy products, the buffalo ricotta sample showed the highest inhibition of ROS release. Furthermore, supported by DPPH radical scavenging assay results (see Supporting Materials), this matrix was selected for further investigation.

The ultrafiltration of G.I. digest of buffalo ricotta cheese revealed that an antioxidant large peptide fraction (F_up_, 60% *w*/*w*), is mainly composed of medium-high molecular weight (1–3 kDa) peptides that are stable to both pepsin cleavage an acidic pH, as previously reported [22,23]. Moreover, the hydrolysis process is deeply affected by the specific technological process used for the ricotta manufacturing. High temperature and rennet adding in addition to high fat content (22% *w*/*w*), cause stable interactions between milk proteins [15], thus reducing the availability to the proteolysis by digestive enzymes.

LC-MS/MS analysis of F_up_ revealed an abundant β-lactoglobulin-derived peptide. Usually di- or tri-peptides are transferred to the basolateral side of the intestinal enterocytes and, thus, absorbed the blood circulation, whereas larger polypeptides are characterized by a low bioavailability, which can determine their accumulation in the gastrointestinal tract, suggesting a local bioactivity [9]. For these reasons, in the present work the potential intestinal protection of BRP on the intestinal epithelial cell line treated with H_2_O_2_ was evaluated. The roles of oxidative stress and the oxidant/antioxidant balance in IBD development have recently received increasing attention. Various gastrointestinal pathological conditions, including gastroduodenal ulcers, malignancies, and irritable bowel disease, arise in part from oxidative stress [2,3,4].

Our data indicated that BRP protects cells from oxidative stress both by inhibiting ROS release and by increasing an antioxidant response, such as NQO1, HO-1, and SOD expression by the activation of the Keap1-Nrf2-ARE pathway, an important antioxidant defense mechanism for cells [21]. Under normal conditions, Keap1 (Kelch-like ECH-associated protein 1) acts as a sensor molecule of oxidative and electrophilic stresses, and it accelerates Nrf2 degradation by proteasomes, thus preventing the hyperexpression of Nrf2 target genes [24]. However, in response to oxidative stress, Nrf2 dissociates from Keap1 and translocates to the nucleus, where it controls the cellular oxidant level and oxidant signaling by regulating the expression of three groups of ARE-dependent genes (antioxidant response element): drug metabolizing enzymes/transporters, oxidant signaling proteins, and antioxidant enzymes/proteins [17,25]. Nrf2 activation regulates antioxidant and cytoprotective enzyme expression, including HO-1 and NQO1. These enzymes are both involved in cellular defense against inflammation and oxidative stress. HO-1 is a cytoprotective enzyme that catalytically degrades heme into biliveridin and iron, producing carbon monoxide (CO) as a byproduct [26]. Induction of HO-1 plays a fundamental role in maintaining cellular homeostasis during inflammation. NQO1 is a highly inducible protein under a variety of stress responses, including oxidative stress. NQO1 is an antioxidant flavoprotein that is able to scavenge ROS [27]. This enzyme is extremely effective at catalyzing the two-electron mediated reduction of quinones to hydroquinones, which is commonly proposed as a mechanism of detoxification. Another enzyme involved in the oxidative stress is SOD. The enzyme SOD neutralizes O_2_ by transforming it into hydrogen peroxide and a hydroxyl radical [28]. Through its activity, SOD enzyme controls the levels of a variety of ROS and reactive nitrogen species, thus both limiting the potential toxicity of these molecules and controlling broad aspects of cellular life that are regulated by their signaling functions [29]. The increase of all these anti-oxidant factors in IEC-6 during oxidative stress conditions contribute to the BRP anti-oxidant effects. BRP could act as indirect inhibitor of Keap1-Nrf2 interaction. In detail, under oxidative stress conditions, carbonyl groups can be generated by side chain oxidation, such as allysine from lysine, 2-amino-3-ketobutyric acid from threonine, and 2-pyrollidone from proline. Aminoacid modification by oxidation produces electrophilic species which form covalent adducts with the sulfhydryl groups of cysteine residues in Keap1, causing the dissociation of Nrf2 from its inactive complex with the repressor protein and its translocation into the nucleus. The antioxidant activity of BRP could also be ascribed to its primary structure. Tyrosine, for instance, is one of the preferred targets for ROS attack [30]. Moreover, the two proline residues of the BRP sequence are particularly important as, in oxidative stress conditions, they can form stable free radical adducts generating hydroxyproline derivatives [31].

## 4. Materials and Methods

### 4.1. In Vitro Gastrointestinal Digestion of Buffalo Ricotta Cheese

Buffalo-milk dairy samples were kindly donated by the San Salvatore Dairy Factory (Capaccio, SA, Campania, Italy). The procedure was performed according to Tenore et al. [32], with slight modification. G.I. digestion was distinguished into gastric and duodenal digestive steps. Briefly, the lyophilized samples were solubilized in deionized water to pH = 2. The mixtures were incubated with pepsin at 37 °C for 2 h and the reaction was stopped by heating the solution at 95 °C for 15 min. After the gastric digestion, the pancreatic digestion was simulated as follows: the digests were incubated in a solution of HCOONH_4_ 10 mM to pH 7.5 and then incubated with pancreatin, chymotrypsin, and bile salts at 37 °C for 2 h; then, the reaction was stopped bringing the solution to pH = 2. A preliminary filtration was carried out for the intestinal digests using filters with 3000 NMWL (Amicon^®^ Ultra-4 3K, Merck Millipore, Tullagreen, Ireland). The devices were centrifuged for 60 min at 6000 rpm at 25 °C (Mikro 220R, Hettich, Kirchlengern, Germany). Finally, to remove salts and sugars, we employed a polymeric reversed phase cartridge. In detail, the peptide fractions were solubilized in distilled water and loaded on a Strata-X 33 µM polymeric reversed phase SPE cartridge (Phenomenex, Bologna, Italy), previously equilibrated in distilled water, then eluted with MeOH 2% *v*/*v* formic acid, and finally lyophilized for 24 h (LyoQuest-55, Telstar Technologies, Terrassa, Spain).

### 4.2. Peptide Fraction Collection from Buffalo Ricotta Cheese

A total of 4 mL of peptide fraction derived from gastrointestinal digestion of buffalo ricotta cheese (1 mg mL^−1^) were loaded on a Microsep Advance Centrifugal Device 1 K (Pall Corporation, Ann Arbor, MI, USA) and were centrifuged for 90 min at 6000 rpm at room temperature. Two peptide fractions with molecular weight < 1 kDa (40% *w*/*w*) and 1–3 kDa (60% *w*/*w*) were collected, filtered through a 0.45 μm pore cellulose membrane (Millipore©), and lyophilized for 24 h (LyoQuest-55, Telstar Technologies, Terrassa, Spain).

### 4.3. LCMS-IT-TOF Analysis of Buffalo Ricotta Fraction with Molecular Weight between 1–3 kDa

The bioactive peptides contained in the most active fraction were analyzed by LC-MS/MS. RP-UHPLC-PDA-ESI-IT-TOF analyses were performed on a Shimadzu Nexera UHPLC system, consisting of a CBM-20A controller, two LC-30AD dual-plunger parallel-flow pumps, a DGU-20A_R5_ degasser, a CTO-20A column oven, an SPD-M20A photo diode array detector, a SIL-30AC autosampler. The UHPLC system was coupled online to an LCMS-IT-TOF mass spectrometer through an ESI source (Shimadzu, Kyoto, Japan). LC-MS data elaboration was performed with LCMSsolution^®^ software (Version 3.50.346, Shimadzu). LC-MS analysis of peptide fractions was carried out on an Aeris^TM^ Peptide 100 × 2.1 mm (100 Å) column (Phenomenex), packed with 1.7 μm core shell particles. The flow rate was 0.5 mL/min and the column oven temperature was set to 60 °C. Injection volume was 1 µL. The following PDA parameters were applied: sampling rate, 40 Hz; detector time constant, 0.160 s; cell temperature, 50 °C. Data acquisition was set in the range of 190–800 nm and chromatograms were monitored at 214 and 220 nm at the maximum absorbance of the compounds of interest. The mobile phase for the analysis of peptide fractions consisted of 0.1% (*v*/*v*) HCOOH/H_2_O (A) and 0.1% (*v*/*v*) HCOOH/ACN (B). Analysis was performed in a gradient elution as follows: 0.01–43.0 min, 0–30% B; 43–45.00 min, 30–95% B; then five minutes for column re-equilibration. MS detection of bioactive peptides was operated in positive ionization mode with the following parameters: detector voltage, 1.60 kV; CDL temperature, 200 °C; block heater temperature, 250 °C; nebulizing gas flow (N_2_), 1.5 L/min; drying gas pressure, 100 kPa. Full scan MS data were acquired in the range of 300–2000 m/z (ion accumulation time, 25 ms; IT, repeat = 3). MS/MS experiments were conducted in data dependent acquisition, precursor ions were acquired in the range of 300–2000 m/z; ion accumulation time, 60 ms; CID energy, 50%; collision gas, 50%; repeat = 1; execution trigger (BPC) intensity, at 40% stop level. To identify peptides sequences MS/MS data file was converted into mzXML format by LCMS solution (Shimadzu), and a free trial of PEAKS 7.5 software (Bioinformatics Solutions Inc., Waterloo, ON, Canada) was employed for sequence determination. The search was performed using a database search tool, by searching against the SwissProt/UniProt database (database Bubalus bubalis release 2017). The most abundant peptide was selected for synthesis and focused in vitro assays.

### 4.4. Solid Phase Synthesis

Nα-Fmoc-protected amino acids, Fmoc-Leu-Wang resin, HBTU, HOAt, DIEA, piperidine, and trifluoroacetic acid were purchased from Iris Biotech (Marktredwitz, Germany). Synthesis of analogue BRP (YVEELKPTPEGDL) was performed according to the solid phase approach using standard Fmoc methodology, by a Biotage Initiator + Alstra automated microwave synthesizer, purified by RP-HPLC, and characterized by direct infusion Fourier transform ion cyclotron resonance MS (FT-ICR).

The peptide was synthesized on a Fmoc-Xaa-Wang resin (0.150 g, 0.69 mmol/g) previously deprotected with 25% piperidine/DMF (1 × 3 min, 1 × 10 min) at room temperature. The resin was then washed with DMF (4 × 4.5 mL). The following protected amino acids were then added on to the resin stepwise. Each coupling reaction was accomplished using a three-fold excess of amino acid with HBTU and HOAt in the presence of DIEA (6 eq.) and were performed at 75 °C for 10 min (2×). After each coupling step, the Fmoc protecting group was removed as described above. The resin was washed with DMF (4 × 4.5 mL) after each coupling and deprotection step. The N-terminal Fmoc group was removed, the resin was washed with DCM (7×), and the peptide was released from the resin with TFA/TIS/H_2_O (90:5:5) for 3 h. The resin was removed by filtration, and the crude peptide was recovered by precipitation with cold anhydrous ethyl ether to give a white powder and then lyophilized.

The crude peptide was purified by RP-HPLC on a semi-preparative C18-bonded silica column (Phenomenex, kinetex 100 Å, 100 × 21.2 mm, 5 µM), with detection at 214 and 220 nm. The flow rate was set to 17 mL/min with mobile phases A: 0.1% TFA in H_2_O *v*/*v* and B: ACN plus 0.1% TFA with a linear gradient starting from 5 to 40% B in 17 min. Analytical purity and retention time of peptide were determined using RP-HPLC-UV and the analogue showed >98% purity when monitored at 214 nm. The exact masses of synthetized peptide was acquired on a SolariX FT-ICR 7T (Bruker Daltonics, Bremen, Germany). The sample was infused at 4 uL/min by a Hamilton syringe. MS detection was operated in positive ionization mode with an ESI Apollo II source with the following parameters: drying temperature, 200 °C; nebulizing gas flow (N_2_), 1 L/min; drying gas pressure, 4 L/min. Full scan MS data were acquired in the range of 100–1500 *m*/*z*, accumulation time, 0.030 ms at 1 M. For MS/MS experiments, precursor ions were isolated with a 3 Da width, with a collision energy of 15 eV; ion accumulation, 150 ms.

### 4.5. Cell Culture

The IEC-6 cell line (CRL-1592) was purchased from the American Type Culture Collection (ATCC, Rockville, MD, USA). The IEC-6 cells originated from normal rat intestinal crypt cells. Cells were cultured using Dulbecco’s modified Eagle’s medium (DMEM, 4 g/L glucose) supplemented with 10% (*v*/*v*) heat-inactivated foetal bovine serum (FBS), 2 mM L-glutamine, 1.5 g/L NaHCO_3_, and 0.1 unit/mL bovine insulin. Cells were used between the 17th and 21st passages for the experiments.

The IEC-6 cells were plated and, after 24 h, were treated with six digesta of buffalo-milk dairy products (yoghurt, scamorza, grana, mozzarella, ricotta, and ice cream) at concentrations of 50–5 µg/mL, with buffalo ricotta fractions (F_up_ and F_down_) at concentrations of 50–5 µg/mL, and BRP at concentrations of 100–1 µM, for 1 h, either alone or in the presence of H_2_O_2_ (1 mM) for different times, as outlined below.

#### 4.5.1. Cell Viability Assay

IEC-6 cells (2 × 10^4^) were plated in 96-well microtiter plates and allowed to adhere for 24 h. Thereafter, cells were exposed to crude digesta of buffalo-milk dairy products (50–5 µg/mL), buffalo ricotta fractions (50–5 µg/mL), and BRP (100–1 µM), for 24 h. Cell viability was then assessed as previously reported using the MTT assay [33]. Briefly, 25 µL of MTT (5 mg/mL) were added and the cells were incubated for 3 h. Thereafter, cells were lysed and the dark blue crystals solubilized with 100 µL of a solution containing 50% (*v*/*v*) *N*,*N*-dimethylformamide, 20% (*w*/*v*) SDS with an adjusted pH of 4.5. The optical density (OD) of each well was measured with a microplate spectrophotometer (Titertek Multiskan MCC/340; Labsystems, Frankfurt, Germany) equipped with a 620 nm filter. IEC-6 viability in response to cell treatment was calculated as: % cellular inhibition = 100 − (OD treated/OD control) × 100.

#### 4.5.2. Measurement of Intracellular ROS Release

ROS levels were evaluated by means of the probe 2′,7′-dichlorofluorescin-diacetate (H_2_DCF-DA) [34]. The IEC-6 cells were plated in 24-well plates (8 × 10^4^ cells/well). After adhesion, cells were treated with all digests of buffalo-milk dairy products (50–5 µg/mL), buffalo ricotta fractions (50–5 µg/mL), and BRP (100–1 µM), for 1 h, either alone or in the presence of H_2_O_2_ at a concentration of 1 mM for a further 1 h. The IEC-6 cells were then collected, washed with phosphate buffered saline (PBS), and then incubated in PBS containing H_2_DCF-DA (10 μM). After 15 min at 37 °C, cell fluorescence was evaluated using a fluorescence-activated cell sorter (FACSscan; Becton Dickinson, Franklin Lakes, NJ, USA) and analyzed with Cell Quest software (Becton Dickinson, Milan, Italy).

#### 4.5.3. Immunofluorescence Analysis for Nuclear Factor-Like 2

IEC-6 cells (2 × 10^5^ cells/well) were seeded on coverslips in a 12-well plate and treated with BRP at concentrations (50 µM) for 1 h in presence of H_2_O_2_ 1 mM for the evaluation of nuclear factor (erythroid-derived 2)-like 2 activation. After, the treatment cells were fixed with 4% paraformaldehyde in PBS and permeabilized with 0.1% saponin in PBS. After blocking with bovine serum albumin (BSA) and PBS, cells were incubated with rabbit anti-Nrf2 antibody (Santa Cruz Biotechnologies, Dallas, TX, USA) for 1 h at 37 °C. The slides were then washed three times with PBS and fluorescein-conjugated secondary antibody (FITC) was added for 1 h. 4′,6-diamidine-2′-phenylindole dihydrochloride (DAPI) was used for the counterstaining of nuclei. Lastly, coverslips were mounted in mounting medium and fluorescent images were taken under a laser confocal microscope (Leica TCS SP5, Leica, Wetzalar, Germany) as previously reported [35].

#### 4.5.4. Measurement of HO-1, NQO1 and SOD Expression

IEC-6 cells were plated into 96-well plates (1 × 10^4^ cells/well), allowed to adhere, and treated with BRP (100–1 µM) for 1 h, either alone or in presence of H_2_O_2_ (1 mM) for 1 h. The IEC-6 cells were then collected, washed with PBS, then incubated in fixing solution for 20 min and then in Fix Perm solution for 30 min. Anti-HO-1 (sc-10789, Santa Cruz Biotechnologies, Dallas, TX, USA), anti-NQO-1 (sc-376023, Santa Cruz Biotechnologies), or anti-SOD (sc-30080, Santa Cruz Biotechnologies) antibodies were then added for a further 30 min. The cells were then treated with the secondary antibody, in Fix solution, and cell fluorescence was evaluated using a fluorescence-activated cell sorter (FACSscan; Becton Dickinson, Milan, Italy) and analyzed with Cell Quest software (Becton Dickinson, Milan, Italy) as previously reported [36].

#### 4.5.5. Data Analysis

Data are reported as mean ± standard error mean (s.e.m.) values of at least three independent experiments, each completed in triplicate. Statistical analysis was performed by the analysis of variance test, and multiple comparisons were made by Bonferroni’s test. A *p* value less than 0.05 was considered significant.

## 5. Conclusions

In this work, antioxidant properties of buffalo-milk dairy products were evaluated. In particular, the attention focused on bioaccessible peptides released during simulated gastrointestinal digestion. Among all the tested dairy products, buffalo ricotta digest has more inhibited ROS release in H_2_O_2_-treated IEC-6 cells. The abundant BRP (YVEELKPTPEGDL), identified as β-lactoglobulin residue (f:60–72), showed significant effects in reducing the oxidative cellular stress, both inhibiting ROS release in H_2_O_2_-treated IEC-6 cells and increasing an antioxidant response, as Nrf2 pathway activation and cytoprotective enzymes expression, such as HO-1, NQO1, and SOD.

Considering the role of oxidative stress both in directly affecting intestinal homeostasis and in activating the pro-inflammatory process, through activation of several regulatory proteins in the tissue [37], anti-oxidant agents could have a potential in the treatment in various gastrointestinal disease. The results indicate how buffalo milk could be an important source of healthy compounds, as well as buffalo ricotta cheese could be considered for formulation of functional and personalized foods, which could be enriched with other natural phytochemical extracts, such as polyphenols [38,39], in order to improve and maintain the state of wellness and to prevent the onset of some gastrointestinal pathologies.

## Supplementary Materials

Supplementary materials can be found at http://www.mdpi.com/1422-0067/19/7/1955/s1.
