# Supplementary material for: Antioxidant Properties of Buffalo-Milk Dairy Products: A β-Lg Peptide Released after Gastrointestinal Digestion of Buffalo Ricotta Cheese Reduces Oxidative Stress in Intestinal Epithelial Cells
Source: Int J Mol Sci. 2018 Jul 4;19(7):1955. doi: 10.3390/ijms19071955 (PMC6073531; doi:10.3390/ijms19071955)
Supplement: Supplementary file 1 [file ijms-19-01955-s001.pdf]

Supporting Material for the manuscript:

Antioxidant Properties of Buffalo-Milk Dairy Products: A β-Lg Peptide Released after Gastrointestinal Digestion of Buffalo Ricotta Cheese Reduces Oxidative Stress in Intestinal Epithelial Cells

S1. DPPH• Radical Scavenging Activity

The DPPH• scavenging activities were determined according to the method previously described by García-Moreno et al. (2014), with some minor modifications. Briefly, a volume of 100 µL (concentration range 0.3–10.0 mg mL^{-1}) of each sample solution was added to 100 µL of DPPH• radical (5×10^{-5} M) in methanol. The mixture was briefly sonicated then left to react for 30 minutes in the dark at room temperature. Then, the absorbance of the reaction mixture was measured at 517 nm. A control solution was prepared by diluting the DPPH solution with methanol in a 1:1 ratio ($A_{DPPH\_control}$). EC50 values were measured as the sample concentration (mg/mL) giving a 50% decrease of the DPPH radical initial concentration and were calculated according to Equation (1):

$$\textbf{DPPH inhibition \%} = [(A_{DPPH\_control} - A_{DPPH\_sample})/A_{DPPH\_control}]\times 100 \quad (1)$$

Table S1. Radical scavenging activity (DPPH• test) of intestinal digesta of buffalo-milk dairy products.

Buffalo-milk dairy products#	EC_{50}*	Equation	R^2
Ricotta	1.91	y = 6.6919x – 1.4372	0.9975
Grana	2.13	y = 6.037x – 0.9197	0.9777
Yogurt	2.20	y = 5.677x – 0.6423	0.9816
Ice Cream	3.93	y = 13.808x – 2.9734	0.9972
Mozzarella	4.16	y = 16.168x – 3.9204	0.9927
Scamorza	4.26	y = 11.460x – 1.4731	0.9886

References

García-Moreno, P.J.; Batista, I.; Pires, C.; Bandarra, N.M.; Espejo-Carpioa, F.J.; Guadix, A.; Guadix, E.M. Antioxidant activity of protein hydrolysates obtained from discarded Mediterranean fish species. *Food Res Int.* **2014**, *65*, 469-476.
